# Editorial: Systems Biology and the Challenge of Deciphering the Metabolic Mechanisms Underlying Cancer

**DOI:** 10.3389/fphys.2017.00537

**Published:** 2017-07-28

**Authors:** Osbaldo Resendis-Antonio, Christian Diener

**Affiliations:** ^1^Coordinación de la Investigación Científica, Red de Apoyo a la Investigación, UNAM Mexico City, Mexico; ^2^Human Systems Biology Laboratory, INMEGEN Mexico City, Mexico

**Keywords:** systems biology, cancer, modeling and simulation, metabolic modeling, dynamical systems

Cancer is one of the major causes of mortality worldwide. One of the particular challenges in battling the disease is that cancers manifest in many different forms each with their own specific genotype and phenotype. Specifically, there is a large variety of genetic and metabolic strategies that cancers employ in order ensure proliferation, metastasis and escape from the immune system of the host. Understanding this mixture of common and specific alterations pose a particular challenge for Science as there are many scales on which to study the disease, ranging from metabolic mechanisms common to all cancers to patient-specific alteration affecting treatment. The intrinsic complexity is astonishing and in order to defeat the disease, we still have a long road to travel. With this purpose in mind, it is crucial to propose new quantitative schemes to gain a better understanding of the mechanisms that underlie modern cancer treatments. Here, Systems Biology approaches have the potential to characterize the metabolic and regulatory mechanisms that support the cancer phenotype and may provide new hypotheses that can cut down the malignant phenotype in clinical treatments. In this context, the works presented in this Research Topic and EBook paint a representative picture of the research landscape and address cancer on various levels of details and mechanisms.

One of the most studied alterations in cancer is the Warburg effect, a switch toward aerobic glycolysis, marked by lactate secretion and a decreased entry of glycolytic intermediates into the citric acid cycle even with an excess of oxygen. Using kinetic models of glycolysis Molavian et al. show how large amounts of oxidative stress byproducts make aerobic glycolysis favorable and Marín-Hernández et al. identify efficient knockout strategies for the increased aerobic cancer glycolysis. Addressing the question which regulatory events may induce the Warburg effect Beltran-Anaya et al. review the impact on non-coding RNAs on glycolysis and related pathways. Those studies are complemented by a set of cancer type-specific works where the inclusion of specific metabolic pathways and transcriptional regulation gives additional perspectives. Roy and Finley use a detailed kinetic model of glycolysis, glutaminolysis, tricarboxylic acid cycle and the pentose phosphate pathway in KRAS-mediated pancreatic cancer in order to reproduce known large-scale knockdown experiments and suggest novel targets to combat pancreatic cancer and Enciso et al. employ a Boolean model to describe a loss of intercellular communication in the molecular regulatory network involved in the development of Acute Lymphoblastic Leukemia. One of the challenges in studying a disease as diverse as cancer is the integration of novel large-scale data in order to increase our understanding of the etiology and progression of the disease, identify proper biomarkers to diagnosis, and promote computational models with higher capacities to predict clinical outcomes in personalized medicine. In this context,Shin et al. review how metabolomics data have helped to characterize particular metabolic mechanisms in the development of head and neck cancers whereas Contreras et al. review the interplay between the microbiota profile and the development of several subtypes of cancer. In our own study (Diener and Resendis-Antonio) we show how large-scale genomic data sets from cell lines and cancer biopsies can be combined in order to improve knowledge about the phenotype of individual biopsies and how this strategy can unravel individual metabolic alterations in a personalized manner.

Additionally, it is important to note that many local alterations in cancer cells take place in tight interplay with the microenvironment and many complex regulatory programs in the surrounding tissues. Thus, one has to be aware that human diseases are not isolated units and one disease can promote or coexist with other diseases in the organism. Altman reviews the interplay between cancer and the circadian cycle in affected cells and its importance in studying metabolic alterations in cancer, whereas Gutierrez Najera et al. suggest a standardization of the phenotypes in neuropsychiatric diseases and their interplay with other diseases such as cancer.

In total, the presented works span a wide variety of approaches to study the metabolic alterations in cancer, showing how methods from Systems Biology can be used in order to formulate more stringent hypotheses about the alterations causing cancer and their potential remedies. Furthermore, there is a clear agreement that any metabolic modeling approach has to be combined with experimental data obtained from various sources. This imposes large possibilities but also challenges for the coming years. As more and larger data sets, sometimes spanning hundreds of thousands of samples, are available this creates a large demand for strategies that can create knowledge and optimized treatment suggestions. Systems Biology will play a large role in addressing those challenges and will have to find novel approaches in order to extend its applicability from general to specific models that may address metabolic alterations in a patient- or sample-specific manner. Finally, we perceive that the near future may bring a breakthrough to the health-care sectors by combining new high-throughput technologies, Bioinformatics and Systems Biology to implement Leroy Hood's and Stephen H. Friend's proposal for a precision medicine capable of being predictive, personalized, preventive and participatory.

## Author contributions

All authors listed have made a substantial, direct and intellectual contribution to the work, and approved it for publication.

### Conflict of interest statement

The authors declare that the research was conducted in the absence of any commercial or financial relationships that could be construed as a potential conflict of interest.

